# Oxalic Diathesis—Oxygenated Bitters

**Published:** 1853-03

**Authors:** Ira Warren

**Affiliations:** Winter Place, Boston


From the Boston Medical and Surgical Journal.

*Oxalic Diathesis—Oxygenated Bitters.*

*To the Editor of the Boston Medical and Surgical Journal.*—Dear Sir,—Dyspeptic invalids often put the inquiry to you, doubtless, as they do to other physicians—"Will it do me any good to take the Oxygenated Bitters?" I propose to make a suggestion as to the class of patients who are benefited by this popular nostrum, and to state what I suppose to be the cause of its usefulness. If in doing this, I can withdraw it, even in the minds of a few, from the list of the secret agencies of hocus-pocus, and transfer it to that of comprehended therapeutic remedies, acting upon rational principles, something will be done, worth, at least, the trouble of tracing these lines.

There is a large class of dyspeptic patients who suffer greatly with depression of spirits They generally have the dark and dingy look of the face which indicates functional derangement of the liver. They are usually emaciated, nervous, hypochondriacal, fearing consumption. They are iritable in temper, seem incapable of exerting themselves, and are exhausted by an excessive secretion of urea. The urine of such persons is always acid, and *loaded with crystals of oxalate of lime.*

I find among the numerous persons I am treating for diseases of the air-passages, that many of them suffer with depression of spirits; and these all have a deposit of oxalate of lime in the urine. This fact, which has been observed by others, has been explained by supposing that the imperfect oxygenation of carbon in inflamed respiratory organs, is vicariously effected in the capillaries of the kidneys—oxalic acid ($C_2 O_2$) instead of carbonic acid ($C O_2$) being the result.

There is another class of persons who suffer much from the oxalic diathesis. It consists of lawyers, clergymen, statesmen, and, in general, those who labor hard mentally with but little bodily exercise, and who have a great weight of care resting upon them.

The crystals of oxalate of lime are octahedral in form; and, in the field of a good microscope, are beautiful objects of inspection. To obtain them, take a portion of urina sanguinis, and let it stand till a deposit takes place. Pour off the upper portion of the urine, put a part of the remainder in a watch-glass, and gently heat it over a lamp. The heat will cause a deposit of the crystals.

The proper treatment of the form of disease here described—a treatment to which it generally yields—is the following:—A careful regulation of the diet, and out-door exercise, with the use of small doses of blue pill, and nitric acid (which is better), mixed with the compound infusion of gentian.

The main part of the treatment is the acid; and I am confident—though I am not aware that any chemical analysis has been made—that the "Oxygenated Bitters" are composed chiefly either of nitric acid, or nitro-hydrochloric acid in the form of aqua regia. It is this principal component, I have little doubt, which has given this nostrum its success in the form of dyspepsia here spoken of, and which has brought so many certificates to the proprietors from that class of persons, including statesmen and others, whom I have mentioned as having oxalate of lime in their urine.

*Winter Place, Boston, February*, 1853. IRA WARREN.

